# Atypical Tibial Fracture Following Chronic Bisphosphonate Use: A Case Report and Review of the Literature

**DOI:** 10.7759/cureus.73165

**Published:** 2024-11-06

**Authors:** Hector Sanchez-Fernandez, Jose I Acosta Julbe, José A Rosario Gonzalez, Sebastian E Frontera, Norman Ramírez, Pedro Reyes Martinez

**Affiliations:** 1 Orthopaedic Surgery, University of Puerto Rico, Medical Sciences Campus, San Juan, PRI; 2 Orthopaedic Surgery, University of Puerto Rico, Medical Sciences Campus, San Juan, USA; 3 School of Medicine, University of Puerto Rico, Medical Sciences Campus, San Juan, PRI; 4 Research, University of Puerto Rico, Medical Sciences Campus, San Juan, USA; 5 Pediatric Orthopedic Surgery, Mayagüez Medical Center, Mayagüez, USA

**Keywords:** atypical femur fracture, atypical fracture, bisphosphonates, intramedullary nail, tibial shaft fracture

## Abstract

Atypical fractures are diagnosed when an otherwise normal force causes a fracture in a patient using bisphosphonates chronically. These occur most commonly in the subtrochanteric region of the femur. Yet, the literature on atypical tibial shaft fractures is scarce. We describe a case of a 60-year-old Hispanic female who presented with an atypical open tibial fracture with a seven-year history of bisphosphonate use, successfully managed with intramedullary nailing.

## Introduction

Osteoporosis is the most common chronic bone disease, occurring at a higher age-adjusted prevalence in Hispanic women compared to non-Hispanic white women [1,2]. It causes decreased bone mass, making patients susceptible to low-energy fragility fractures [3]. These occur at a rate of 9 million per year worldwide and present in 33% of women over 50 years old [1].

The most common treatment is bisphosphonates, designed to slow down bone loss by inhibiting osteoclast activity [4]. Side effects of these medications include gastrointestinal and flu-like symptoms, hypocalcemia, musculoskeletal pain, impaired ocular problems, cutaneous reactions, and atypical femoral fractures [5]. Extended use of bisphosphonates also makes patients susceptible to atypical long bone fractures, characterized on radiographs by endosteal thickening and a cortical spike [6,7]. Femurs are the most affected long bone in these patients [8]. Shane et al. found that atypical femur fractures occur at a rate of 100 cases per 100,000 person-years when accounting for chronic bisphosphonate exposure [9].

The second most affected bone is the tibia [10]. Yet, there is a lack of literature on atypical tibial shaft fractures secondary to chronic bisphosphonate use. A few cases have been reported, but no Hispanic cases have been described in the literature [10-15]. This underrepresentation is likely due to under-reporting and a lack of comprehensive registries documenting these cases rather than differences in bone mineral density threshold or racial factors. We aim to report the case of a 60-year-old Hispanic female with a history of osteoporosis managed with chronic bisphosphonates for 7 years who presented with an open atypical tibial shaft fracture.

## Case presentation

This is the case of a 60-year-old Hispanic female who presented to the emergency department after experiencing a fall from a standing height. Upon presentation, she denied any previous history of fractures and had no systemic symptoms. She has a past medical history of hypertension and osteopenia and rejected the use of alcohol, drugs, or any illicit substance. Her mother had a history of osteoporosis. Her past surgical history includes a hysterectomy with unilateral salpingo-oophorectomy due to a preoperative diagnosis of endometriosis around 20 years before sustaining this fracture. Six years after the hysterectomy, she started hormone replacement therapy, taking Premarin 0.45 milligrams (mg) per os (PO) once a day. Seven years before the injury, her last dual-energy X-ray absorptiometry (DEXA) bone scan performed at the left femoral neck displayed a T-score of -2.3, confirming the diagnosis of osteopenia. She was consequently started on alendronate 70 mg PO once weekly.

**Figure 1:**
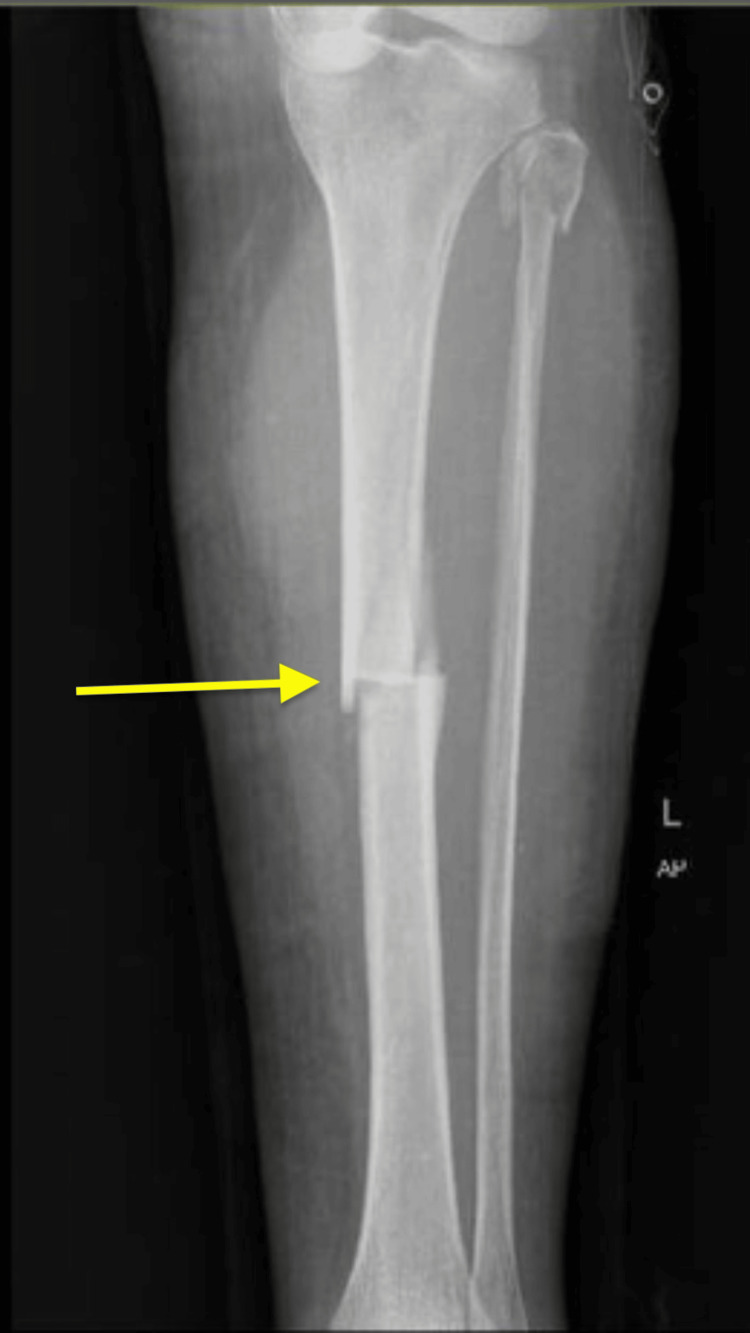
AP XR view of the left tibia shaft fracture showing a displaced fracture in the anterior plane with a posterior spike and anterior endosteal thickening Minimal comminutions are present at the tibia shaft and fibular head fractures. AP: anteroposterior; XR: X-ray

**Figure 2:**
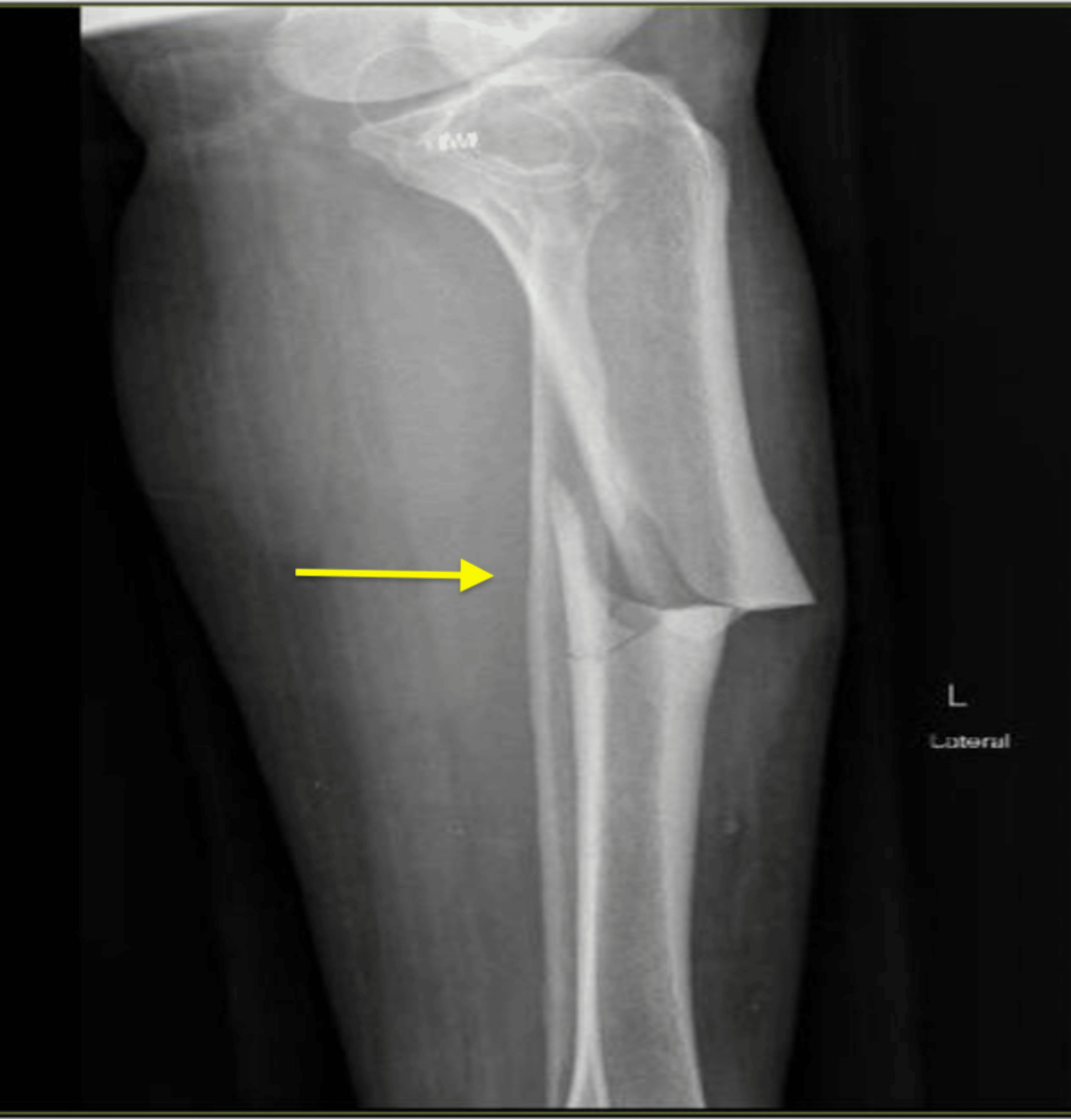
Lateral XR view of the left tibia shaft fracture showing a displaced fracture in the anterior plane with a posterior spike and anterior endosteal thickening Minimal comminutions are present at the tibia shaft and fibular head fractures. XR: X-ray

Physical exam findings showed an alert and oriented female with an open left tibial shaft fracture type I Gustilo classification. No active bleeding was noted, the compartments were soft and depressible, and the patient was neurovascularly intact distally. Laboratory findings included calcium at 9.2 mg/deciliters (dL) (reference range: 9-10.5 mg/dL), phosphate at 3.1 mg/dL (reference range: 2.5-4.5 mg/dL), parathyroid hormone at 37 pg/mL (reference range: 15-65 pg/mL), and 25-hydroxy vitamin D at 30 ng/mL (reference range: 30-100 ng/mL), all within normal ranges. X-rays of the left tibia showed a displaced transverse fracture with anterior cortical thickening and a posterior spike seen on the lateral view, consistent with an atypical tibial fracture. Additionally, minimal comminution was seen in the tibial and fibular head fractures (Figures 1-2). To exclude fracture of other bone involvement and impending fractures in the lower extremities, radiographs of the ipsilateral femur, contralateral femur, and contralateral tibia were performed without atypical findings. The tibial fracture was closely reduced and splinted with a long posterior splint until surgical management.

**Figure 3:**
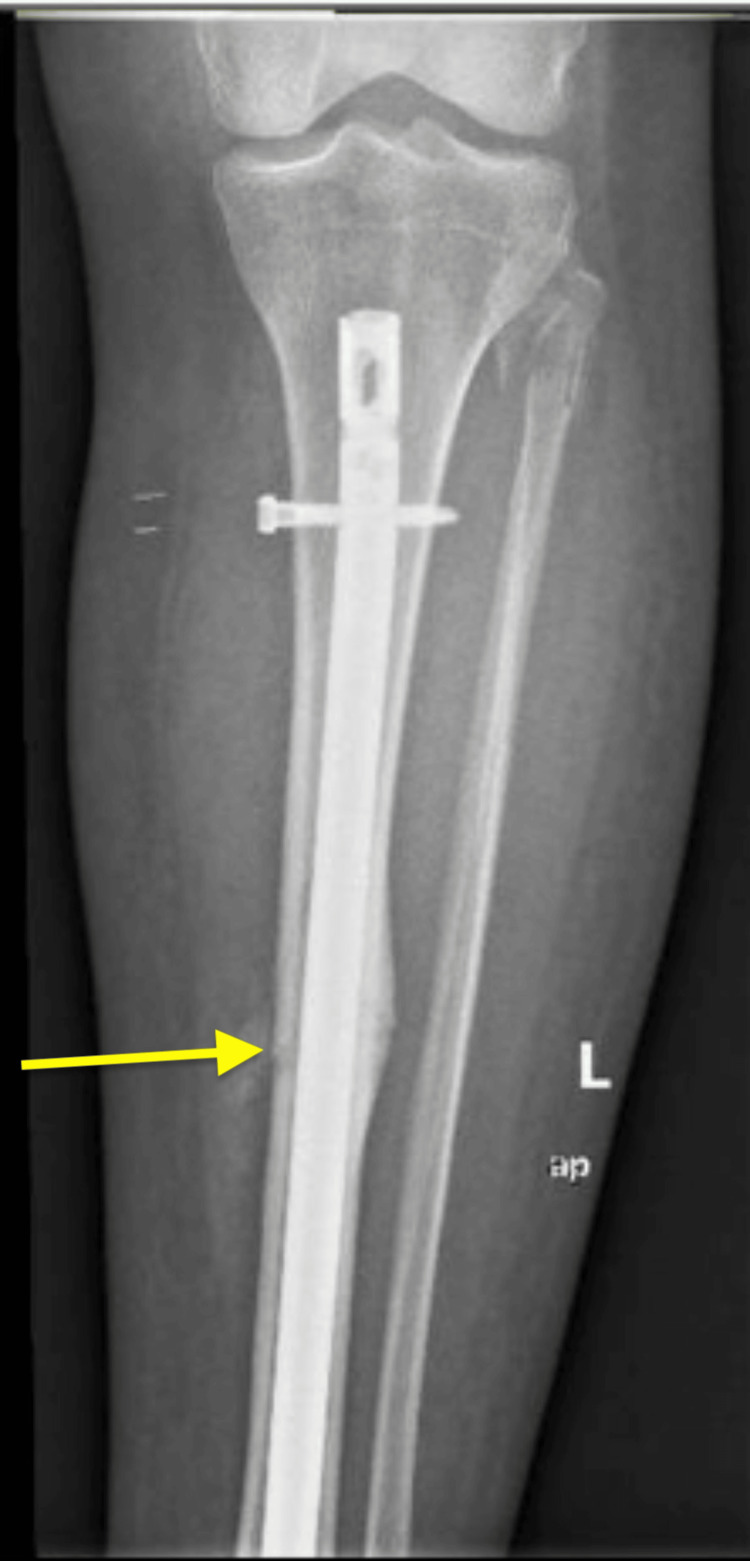
AP XR views of the left tibiofibula following IMN AP: anteroposterior; XR: X-ray; IMN: intramedullary nailing

**Figure 4:**
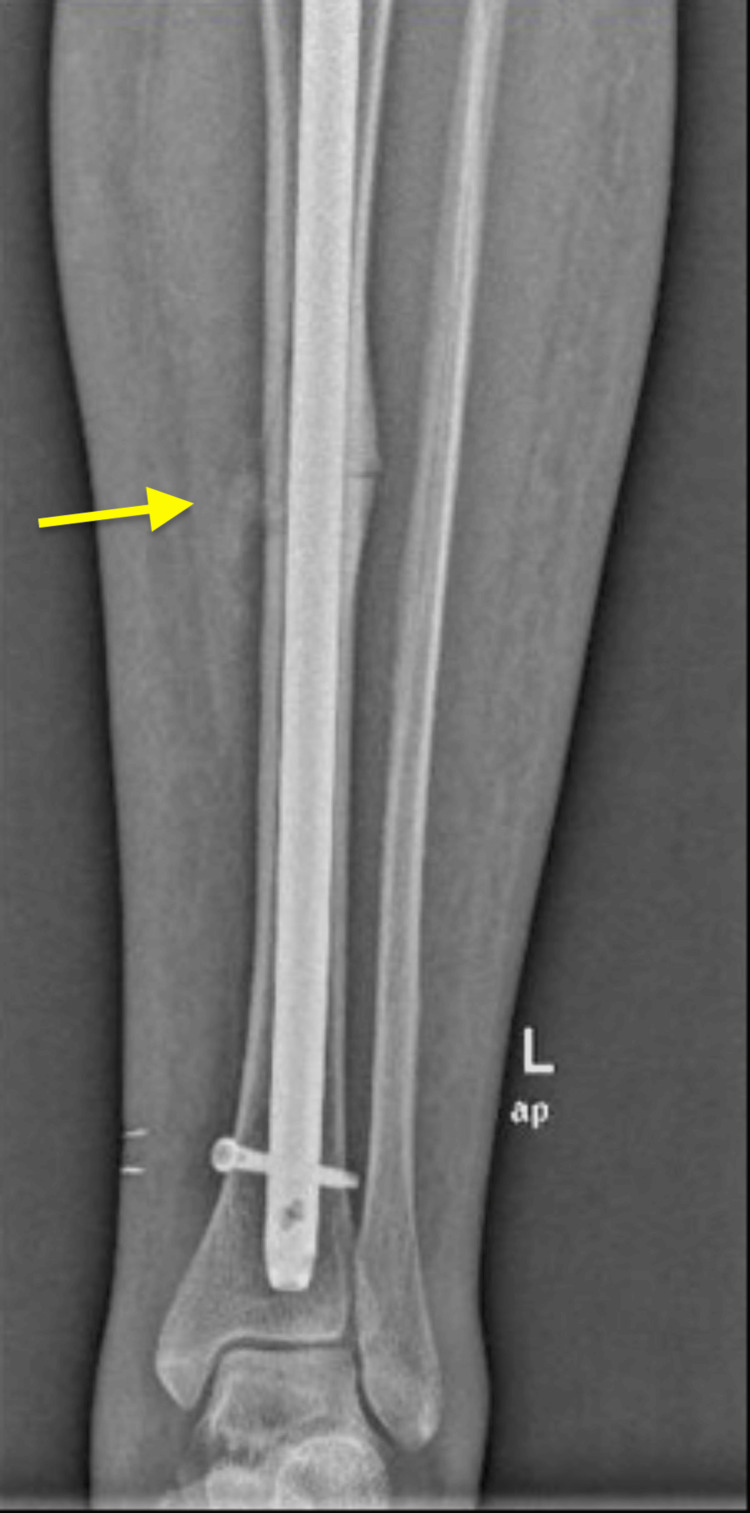
AP XR views of the left tibiofibula following IMN AP: anteroposterior; XR: X-ray; IMN: intramedullary nailing

**Figure 5:**
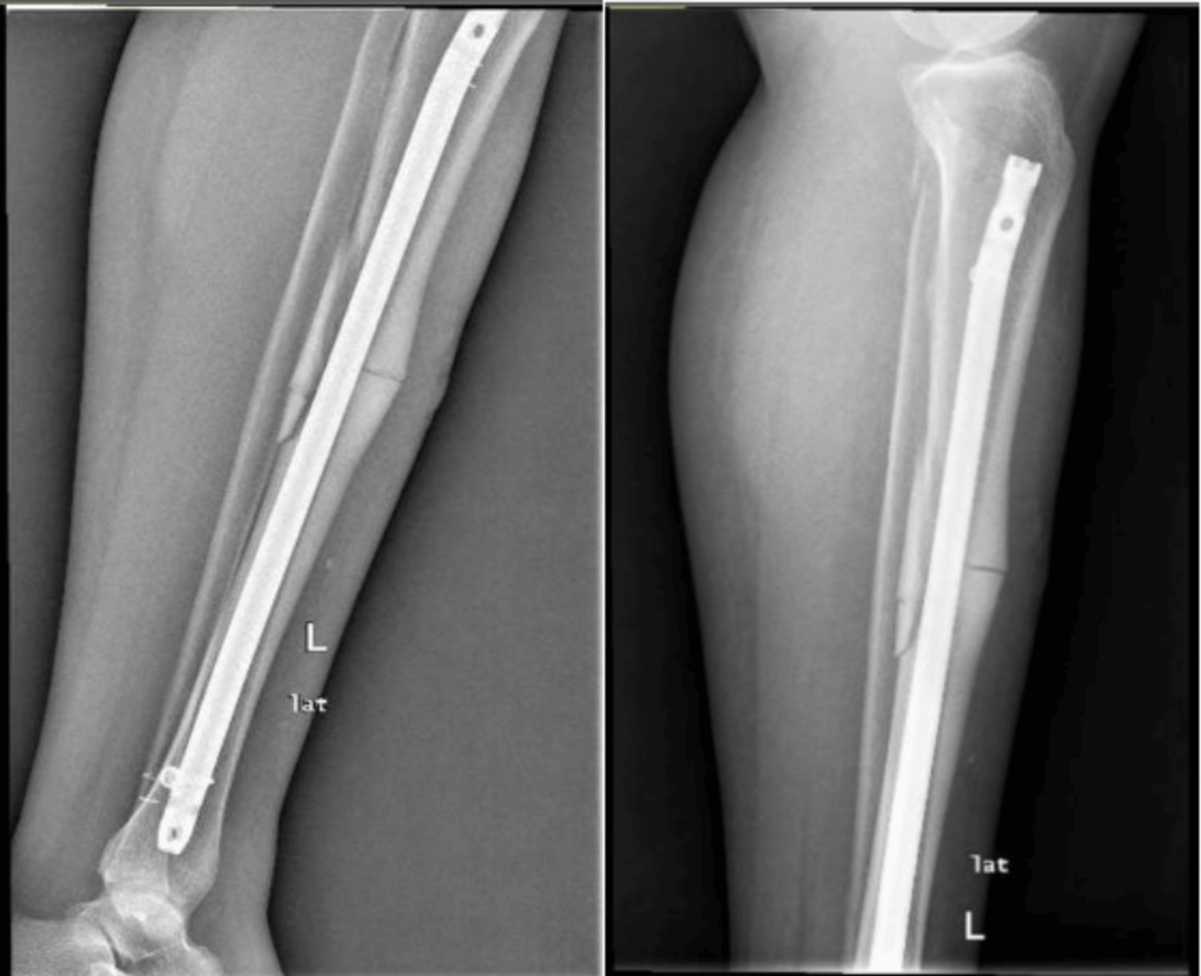
Lateral XR views of the left tibia following IMN XR: X-ray; IMN: intramedullary nail fixation

After discussing treatment alternatives and obtaining her consent, the patient underwent intramedullary nailing (IMN) without any intraoperative complications (Figures 3-5). The patient was discharged when medically optimized, and there was no evidence of infection. Alendronate was discontinued and replaced by 5000 IU of Vitamin D3 PO daily and 1200 mg of calcium PO daily. In addition, we recommended close follow-up with endocrinology.

**Figure 6:**
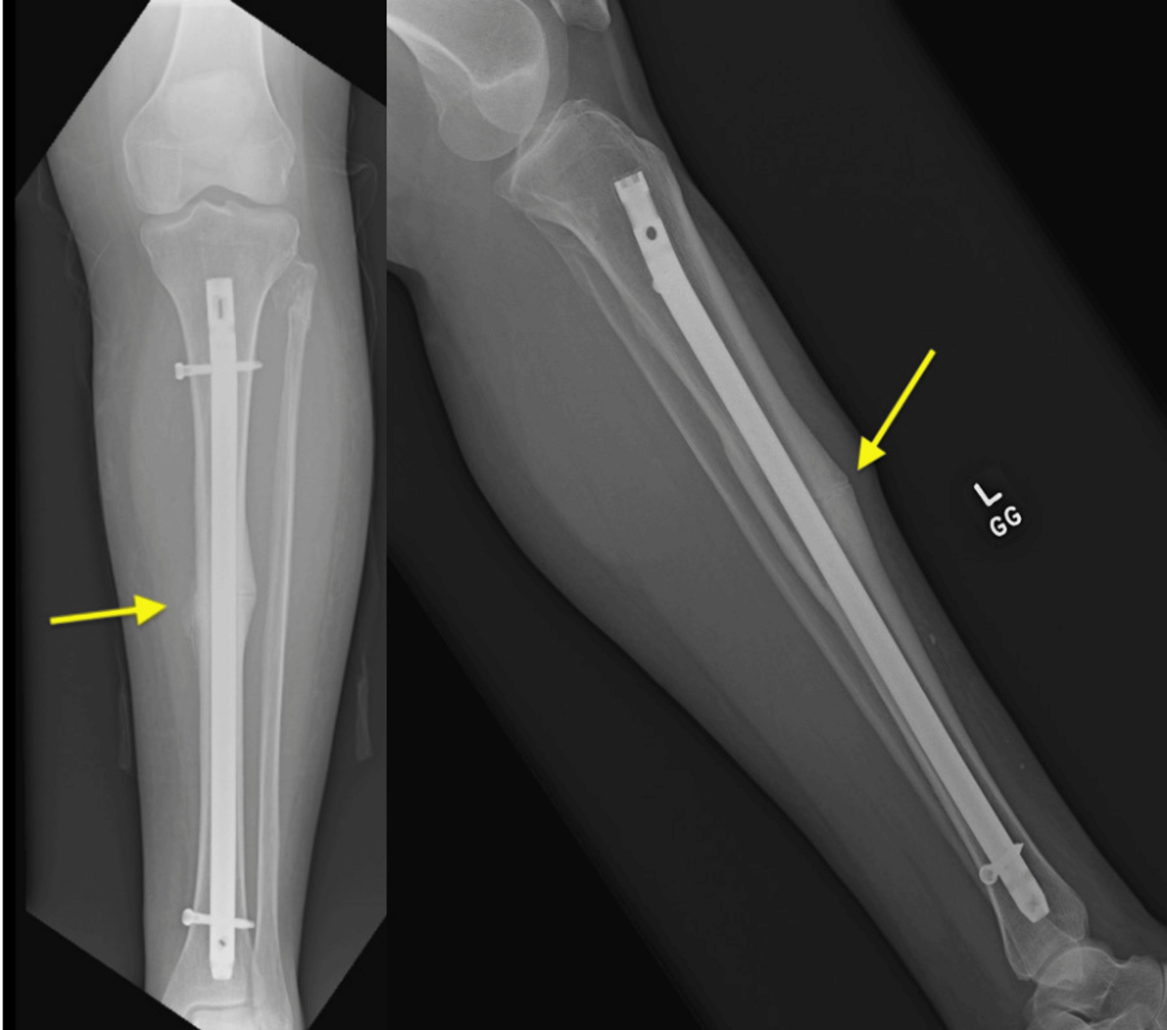
The X-rays depict the anteroposterior (left) and lateral (right) views of the tibia with an IMN in place The images show a tibial shaft fracture stabilized by the IMN, which extends from the proximal tibia to the distal segment. The yellow arrows highlight the fracture site, demonstrating callus formation, alignment, and positioning postoperatively. IMN: intramedullary nailing

At the three-month follow-up visit, radiographs showed early signs of callus formation at the fracture site, indicating the initial stages of bone healing (Figure 6).

Soft tissue healing was adequate, with the wound fully epithelialized and no signs of infection or complications. The patient was able to bear weight without pain or discomfort. At the six-month follow-up visit, radiographs demonstrated increased callus formation and bridging of the fracture site, consistent with progressive bone healing. At the two-year visit, the patient remained asymptomatic, with radiographs confirming sustained fracture healing and bone integrity, and she reconvened her activities of daily living.

## Discussion

Tibial shaft fractures are a leading cause of disability worldwide, particularly in low- and middle-income countries [16]. Around 37% are present after motor vehicle accidents, while less than 2% occur secondary to falls from standing height [17]. As presented in our case, atypical tibial shaft fractures have been described as having an anterior cortical thickening in patients undergoing long-term treatment with bisphosphonates [11]. These also commonly present as transverse, non-displaced, and with slight comminution [15,18]. This type of fracture is secondary to bisphosphonates [12]. Donnelly et al. found that the longer the duration of bisphosphonate therapy, the greater the risk of developing atypical long bone fractures [19]. In 2004, Odvina et al. described how bisphosphonate inhibition of osteoclasts may lead to oversuppression of bone turnover, creating a state of “frozen bone” and increased skeletal fragility [20]. We describe a case of a 60-year-old Hispanic woman with a left open atypical tibial shaft fracture secondary to chronic bisphosphonate use.

The use of bisphosphonates, such as alendronate, as the gold standard to reduce the risk of fractures in osteoporotic patients has been well supported in the literature [21-23]. Siris et al. observed an estimated prevalence of around four million women over 45 receiving bisphosphonate treatment [24]. In 2008, Wells et al. demonstrated a statistically significant decrease in osteoporosis-related fractures when patients were treated with oral alendronate [25]. Current guidelines recommend treatment with bisphosphonate for adults with a high risk of fragility fractures, which are identified using the fracture risk assessment tool (FRAX) [26]. According to the National Osteoporosis Foundation Guide, patients with an osteoporotic fracture risk score of 20% or above on the FRAX 10-year fracture risk assessment should receive treatment with alendronate [27]. In our patient, the FRAX 10-year fracture risk score was 20%. Thus, Alendronate treatment was initiated to decrease the risk of fractures.

Despite the effectiveness of bisphosphonates, alternative osteoporosis alternatives are available and should be considered, particularly in cases where long-term bisphosphonate use poses risks. Selective estrogen receptor modulators, such as raloxifene, can reduce vertebral fractures and have additional cardiovascular benefits, although they do not affect non-vertebral fractures [28]. Another option is denosumab, a monoclonal antibody that reduces bone resorption and has shown efficacy in preventing vertebral, non-vertebral, and hip fractures [29]. However, its discontinuation can lead to rapid bone loss, highlighting the need for careful monitoring [29].

Atypical fractures were first described as a complication of chronic bisphosphonate use in 2005, and current literature on the diagnostic criteria of atypical femur fractures (AFF) was released by the American Society of Bone and Mineral Research (ASBMR) in 2010 [9]. However, the ASBMR guidelines make no mention of long bone fractures other than AFF [9]. According to these guidelines, an AFF must have four out of five major features with a possibility of minor features (Table 1) [9].

**Table 1:** ASBMR Task Force case criteria for the definition of atypical femur fractures ASBMR: American Society of Bone and Mineral Research

ASBMR Task Force Case Definition of Atypical Femur Fractures, Revised Criteria	
Major Criteria (4 of 5 must be met)	Minor Criteria (may or may not be met)
Associated with no trauma or minimal trauma, as fall from standing height or less.	Generalized increase in cortical thickness of the femoral diaphysis.
Fracture originates at the lateral cortex and is substantially transverse in its orientation, although it may become oblique as it crosses the medial femur.	Prodromal symptoms such as dull or aching pain in the groin or thigh.
Noncomminuted.	Bilateral incomplete or complete femoral diaphysis fractures.
Complete fractures extend through both cortices and may be associated with a medial spike; incomplete fractures involve only the lateral cortex.	Delayed fracture healing.
Localized periosteal or endosteal thickening on the lateral cortex occurs at the fracture site.	Expressly excluded are femoral neck fractures, intertrochanteric fractures with spiral subtrochanteric extension, pathological fractures associated with primary or metastatic bone tumors, and periprosthetic fractures.

Although these were developed for the femur, our patient's tibial fracture exhibits all the major features with some modifications. First, the tibial fracture was associated with minimal trauma since the fall was from a ground-level height. Atypical femur fractures frequently present from lateral to medial, whereas atypical tibial fractures present from anterior to posterior, suggesting that these follow the natural bowing of these long bones (i.e., varus bowing of the femur shaft and anterior bowing of the tibia) [30]. The fracture is predominantly transverse in orientation and becomes oblique as it reaches the posterior cortex. The fracture had minimal comminution. Lastly, there is extensive cortical thickening from the cortices at the level of the fracture.

There have been a total of eight cases of atypical tibial shaft fractures due to chronic bisphosphonate use [10-15,18,31]. Yet, we describe the first case of an atypical open tibial shaft fracture secondary to chronic bisphosphonate use in a Hispanic female. Compared to other cases, the fracture described in our case exhibited an open and displaced pattern with a posterior spike. While current concepts on managing atypical femoral fractures are well-established, there is a sparsity in the literature describing the management of atypical tibial fractures. Given the similarity with AFF, further research should assess the implementation of the guidelines for femoral fractures in patients presenting with atypical tibial shaft fractures. These include management of the contralateral side depending on the patient’s symptoms: if asymptomatic with no or minimal radiographic changes concerning impending fracture, close surveillance is recommended; if radiographic signs concerning impending fracture (i.e., the presence of the “dreaded black line”), prophylactic IMN is recommended [32]. Lastly, patients without radiographic changes who note consistent thigh pain on the contralateral side either before or after fracture should be considered for management with prophylactic IMN [32].

## Conclusions

This report demonstrates the rare occurrence of an atypical tibial fracture secondary to chronic bisphosphonate use in a Hispanic woman without a previous fracture history. Further research studies should assess the implementation of management guidelines for atypical femoral fractures in patients presenting with atypical tibial shaft fractures.
